# Bilateral endogenous *Fusarium solani* endophthalmitis in a liver-transplanted patient: a case report

**DOI:** 10.1186/1752-1947-8-101

**Published:** 2014-03-24

**Authors:** Jesper Skovlund Jørgensen, Jan Ulrik Prause, Jens Folke Kiilgaard

**Affiliations:** 1 Department of Ophthalmology, Glostrup Hospital, University of Copenhagen, Nordre Ringvej 57, 2600 Glostrup, Denmark; 2 Eye Pathology Institute, Department of Neuroscience and Pharmacology, University of Copenhagen, Frederik V`s vej 11,1, 2100 Copenhagen Denmark

**Keywords:** Basement membrane collagen, Endophthalmitis, *Fusarium solani*, Immunocompromised, Integrin, Liver transplantation

## Abstract

**Introduction:**

Endogenous *Fusarium* endophthalmitis is a rare disease predominantly described in immunocompromised patients often due to leukemia. We report a case of bilateral endogenous *Fusarium solani* endophthalmitis in a liver-transplanted patient.

**Case presentation:**

A 56-year-old Danish Caucasian woman who had undergone two liver transplantations, developed endogenous endophthalmitis of her left eye 10 days after the second liver transplantation. Despite continuous therapy, enucleation of her left eye was eventually necessary; at this point funduscopic examination of her right eye disclosed a white inflammatory plaque at the macula consistent with a fungal infection. Microbiological analysis of vitreous fluid from her enucleated left eye revealed *Fusarium solani*, and light microscopy of her enucleated eye was consistent with *Fusarium* panophthalmitis with massive ingrowth of the fungi in all areas containing basement membrane collagen. Voriconazole was injected intravitreally in her right eye, and intravenous voriconazole was initiated. No subsequent growth in the inflammatory plaque was observed. She died 6 weeks after the endogenous endophthalmitis was diagnosed.

**Conclusions:**

This is the first report of endogenous *Fusarium solani* endophthalmitis in a liver-transplanted patient. Ophthalmologists and physicians dealing with liver transplantation should be aware of the potential for postoperative endophthalmitis due to rare microorganisms, such as *Fusarium solani*.

## Introduction

Fungal endophthalmitis (FE) is a rare disease associated with poor visual outcome [1]. The condition is divided into exogenous FE and endogenous FE, the latter assumed to be caused by a hematogenous spread. *Candida* species are the most frequent pathogens [2]. Endogenous *Fusarium solani* endophthalmitis has been reported in a few patients with leukemia or after bone marrow transplantation [3,4]. Here we describe a bilateral endogenous *Fusarium solani* endophthalmitis in an immunocompromised liver-transplanted patient.

## Case presentation

A 56-year-old Danish Caucasian woman was diagnosed with primary biliary cirrhosis in 2004. A liver transplantation was performed in autumn 2011, and immunosuppressive therapy with cyclosporin A, mycophenolate mofetil and prednisolone was initiated. Liver ischemia and multiple infections complicated her post-transplant period and a re-transplantation was performed 5 weeks later. Again her early post-transplant period was complicated by infections. Her blood cultures showed Gram-positive cocci, *Candida albicans* and cytomegalovirus. She was treated with intravenous meropenem (3 × 1g/day), linezolid (2 × 600mg/day), micafungin (1 × 100mg/day) and ganciclovir (2 × 75mg/day). She developed conjunctival hyperemia, corneal edema, exudates and inflammatory cells in the anterior chamber of her left eye 10 days after re-transplantation. No corneal infiltrates or ulcers were found. Due to inflammation in the anterior chamber, the visualization of the vitreous and the retina was impaired. Information of subjective symptoms, like ocular pain, and visual acuity was not available in this sedated and intubated patient. Slit lamp examination and indirect ophthalmoscopy of her right eye did not reveal any pathology.

Endogenous endophthalmitis from either Gram-positive cocci or *Candida albicans* was suspected, but her overall condition did not favor sampling from the vitreous and this was not performed. The intravenous antibacterial and antifungal treatment was continued, and topical treatment with ciprofloxacin and a dilating agent was added.

At this point her white blood cell count was 8.1 × 10^9^/L and neutrophil cell was 5.5 × 10^9^/L, but within 12 days she became neutropenic and her neutrophil cell count decreased to 0.7 × 10^9^/L.

Additional microbiological cultures from her tracheal aspirate and urine were positive for *Candida albicans* and now also filamentous fungi. Analysis of the tip of a central venous catheter revealed Gram-positive cocci and a filamentary fungus growth.

Because she continuously deteriorated, enucleation of her left eye was performed after 13 days in order to exclude the eye as the source for the continuous infection. At this point funduscopic examination of her right eye disclosed a white inflammatory plaque at the macula consistent with a fungal infection. There was no conjunctival hyperemia or inflammation in the anterior chamber.

Microbiological analysis of the vitreous fluid from her enucleated eye revealed only hyphae, subsequently identified as *Fusarium solani* by polymerase chain reaction. The day after the enucleation 0.1mg of voriconazole was injected intravitreally in her right eye, and intravenous voriconazole (2 × 600mg/day) was initiated. Intravenous treatment with meropenem, linezolid, ganciclovir and micafungin was continued. Afterwards no growth in the inflammatory plaque was observed, and there were no sign of inflammation in the anterior parts of her eye. Since she continued to deteriorate despite intensive care it was decided to end active treatment; she died 8 weeks after the second liver transplantation due to septic shock-related multi-organ failure.

## Discussion

**Figure 1:**
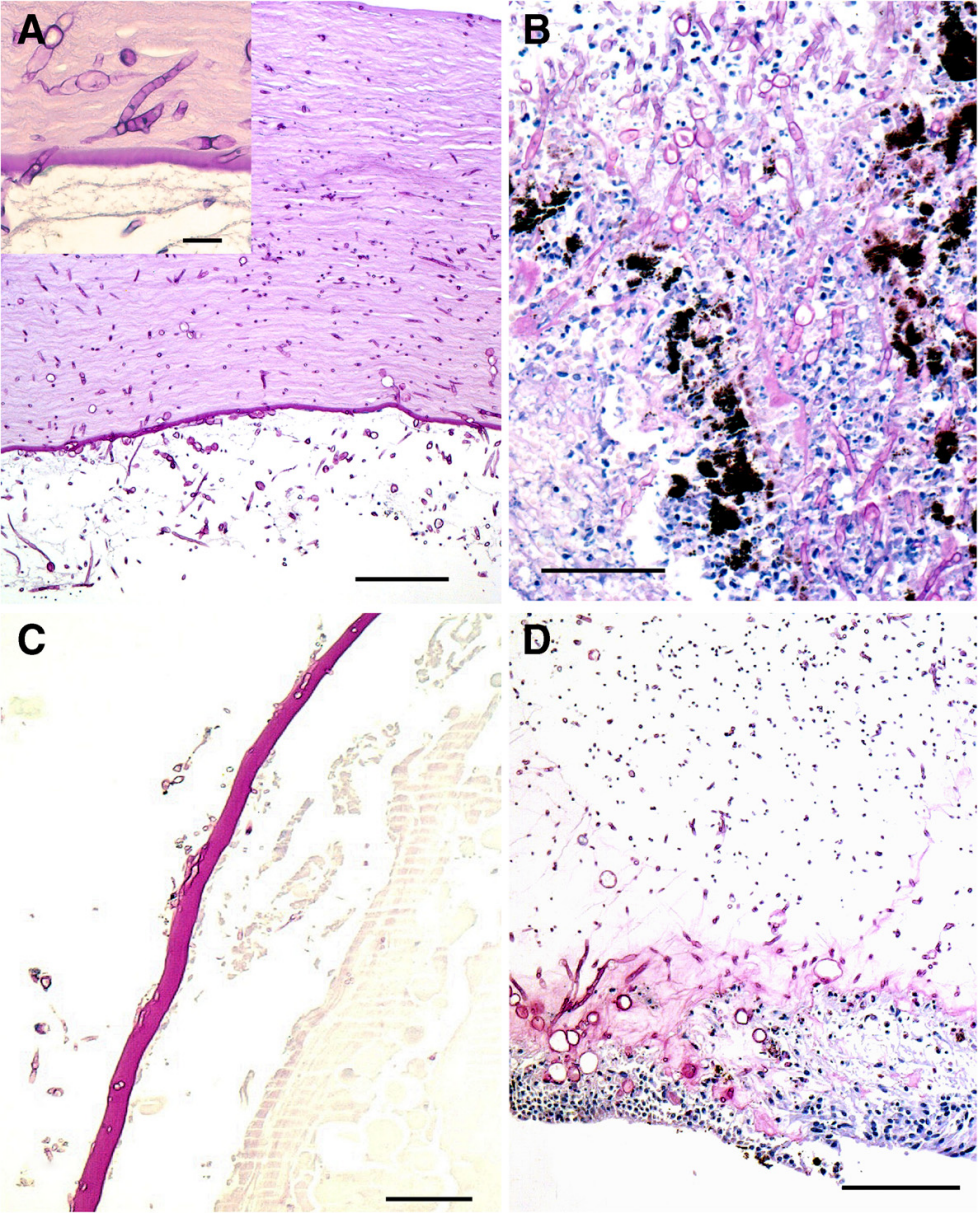
**Micrograph of the enucleated eye with *Fusarium*.** Bars = 100μm, periodic acid–Schiff stains. **(A)** Cornea with hyphae, note their increasing density towards Descemet’s membrane. Insert: higher power of hyphae in Descemet’s membrane. **(B)** Posterior chamber with hyphae extending from the iris and ciliary body. **(C)** Posterior lens capsule infiltrated by hyphae. **(D)** Necrotic retina. Note that the hyphae infiltrate predominantly the internal limiting membrane.

Light microscopy of her enucleated eye showed features of a *Fusarium* panophthalmitis. Her corneal stroma was infiltrated by septated hyphae, increasing in density towards the corneal endothelium, penetrating the Descemet’s membrane (Figure 1A). The anterior chamber was filled with debris and hyphae. The iris and ciliary body were invaded by hyphae that penetrated to the posterior chamber (Figure 1B). Hyphae were found in the anterior aspect of the vitreous and were invading through the lens capsule (Figure 1C). Her sclera, optic nerve, choroid and retinal pigment epithelium were without fungi, however, hyphae were abundant along the internal limiting membrane of the retina (Figure 1D).

The endogenous *Fusarium* endophthalmitis in this eye is characterized by massive ingrowth of the fungi in all areas containing basement membrane collagen, that is, Descemet’s membrane, lens capsule and internal limiting membrane of the retina. There were no fungi in the anterior parts of the cornea including Bowman’s layers, and no clinical signs of fungal keratitis and exogenous endophthalmitis. We might speculate that *Fusarium solani* has an affinity for basement membrane collagen and integrin and therefore the ingrowth of the fungi is most pronounced in these areas. This is supported by results of *in vitro* studies of human corneal epithelial cells indicating that *Fusarium solani* adhesion to epithelial cells is dependent on β1 integrin [5]. A histological study of enucleated human eyes identified that integrins and β1 integrin constitute a part of the internal limiting membrane of the retina [6].

The intraocular infection by *Fusarium solani* in our patient was probably the result of hematogenous dissemination, despite the fact that no blood cultures identified *Fusarium solani*. The source of the infection may have been the tip of the central venous catheter where microbiological analysis revealed a filamentary fungus growth. Colonization of central venous catheter by *Fusarium solani* has been reported previously [7].

## Conclusions

To the best of our knowledge, this is the first case of endogenous *Fusarium solani* endophthalmitis in a liver-transplanted patient; it emphasizes that unusual microbes should be considered a cause of endogenous endophthalmitis in immunocompromised patients. The immunosuppression of the patient was most probably the primary cause for this fulminant case of endophthalmitis. Endogenous endophthalmitis was the first clinically apparent manifestation of disseminated fusariosis in this patient. A vitreous sampling, performed at the time when endophthalmitis was diagnosed, would probably have demonstrated *Fusarium solani*, but this was not carried out.

## Consent

Written informed consent was obtained from relatives of the patient for publication of this case report and accompanying images. A copy of the written consent is available for review by the Editor-in-Chief of this journal.

## Abbreviation

FE: Fungal endophthalmitis.

## Competing interests

The authors declare that they have no competing interests.

## Authors’ contributions

JFK and JSJ were in charge of the ophthalmological care of the patient. JUP performed the histological examination of the eye. JSJ, JFK and JUP wrote the manuscript. All authors read and approved the final manuscript.
